# Exploring clinical chemistry markers in amyotrophic lateral sclerosis: insights into survival and disease trajectories

**DOI:** 10.1007/s00415-024-12774-7

**Published:** 2024-12-12

**Authors:** Ioannis Psychogios, Yihan Hu, Christina Seitz, Emily E. Joyce, Anikó Lovik, Caroline Ingre, Fang Fang

**Affiliations:** 1 Institute of Environmental Medicine, Karolinska Institutet, Stockholm, Sweden; 2 Institute of Psychology, Leiden University, Leiden, The Netherlands; 3 Department of Clinical Neuroscience, Karolinska Institutet, Stockholm, Sweden; 4 Neurology Clinic, Karolinska University Hospital, Stockholm, Sweden; 5 Unit of Integrative Epidemiology, Institute of Environmental Medicine, Karolinska Institutet, Stockholm, Sweden

**Keywords:** ALS, Clinical chemistry, Blood markers, Survival, Prognosis

## Abstract

**Objective:**

Commonly measured clinical chemistry markers might be indicative of survival and disease progression in amyotrophic lateral sclerosis (ALS).

**Methods:**

In a cohort study of 270 ALS patients diagnosed from April 2014 to May 2021 in Stockholm, Sweden, we examined the link between 29 clinical chemistry markers at diagnosis and mortality risk at 6 months, 1 year, and 3 years after diagnosis. Summary variables from exploratory factor analysis (EFA) assessed the markers’ collective impact on survival. We integrated ALS functional rating scale-revised (ALSFRS-R) scores with survival data using a joint latent class model to identify patterns of functional decline. Multinomial logistic regression determined how the EFA-derived factors predicted the decline trajectories post-diagnosis.

**Results:**

Higher levels of total cholesterol, low-density lipoprotein cholesterol (LDL-C), apolipoprotein B, and albumin at diagnosis were linked to lower mortality in ALS patients, while increased neurofilament light chain (NfL), leukocyte count, mean corpuscular volume (MCV), mean corpuscular hemoglobin (MCH), and carbon dioxide (CO_2_) levels indicated higher mortality. The ‘Red blood cell profile’ factor, derived from EFA, emerged as a significant predictor of survival, independent of other prognostic indicators. The joint latent class model identified three distinct patient groups based on functional decline, with ‘Red blood cell profile’ suggesting a lower likelihood of being in the groups with slower progression.

**Conclusion:**

Clinical chemistry markers, including NfL, lipids, albumin, leukocyte count, MCV, MCH, CO_2_, and the ‘Red blood cell profile,’ were associated with ALS survival. As these markers represent broader bodily functions, integrating them in ALS patient care could improve disease management.

**Supplementary Information:**

The online version contains supplementary material available at 10.1007/s00415-024-12774-7.

## Background

Amyotrophic lateral sclerosis (ALS) is a heterogenous neurodegenerative disease characterized by the degeneration of both upper and lower motor neurons, leading to a wide range of clinical symptoms. The disease manifests in different forms, the most common being spinal onset, affecting the limb and trunk muscles, followed by bulbar onset, affecting the oral, facial, and pharyngeal muscles, and respiratory onset, affecting the thoracic muscles [1, 2]. In more than half of patients with ALS, there is some degree of cognitive or behavioral impairment, even if the criteria for dementia are not fulfilled [3–5].

Approximately 10–15% of ALS patients are familial, with the remaining being sporadic [6]. The diagnostic process for ALS is complex and protracted, typically taking around a year from the initial symptom onset [7, 8]. Diagnostic delay can be attributed to delay in seeking medical care, wrong referrals, misdiagnoses, and the necessity to rule out other neurological conditions that mimic ALS [8]. Survival in ALS varies substantially, ranging from only a few months to over 10 years, with a median survival of 2–5 years from symptom onset [9]. Only a minority, about 10%, survive beyond 10 years [10]. Factors such as older age at onset, presence of *C9orf72* repeat expansion, presence of cognitive impairment, and bulbar onset have been linked to a more rapid disease progression [11, 12].

Clinical chemistry measures blood and other fluid components like enzymes, hormones, and lipids, aiding in the diagnosis and management of various diseases [13]. While studies exploring the associations of clinical chemistry markers with outcome of patients with ALS are accumulating, prior studies have mainly focused on specific sets of biomarkers, such as lipids and lipoproteins [14, 15], creatinine and albumin [16, 17], and inflammatory markers [18]. Further, these studies often involved short follow-up periods, without evaluating the synergistic predictive value of these biomarkers.

Management of symptoms in the bodily systems outside of the central nervous system has become increasingly important in ALS, e.g., pharmacological interventions have been proposed targeting hypermetabolism and metabolic disturbances [19] and oxidative stress [20]. A previous clinical trial found that a high-calorie diet extended the survival of patients with ALS [21] whereas another clinical trial showed that curcumin (a natural antioxidant compound) supplementation slightly decreased disease progression [22]. As clinical chemistry markers reflect various bodily functions, it is important to understand the prognostic values of these biomarkers in ALS survival, with the goal of improving personalized treatment and management strategies.

Neurofilaments, part of the cytoskeleton, are a marker of axonal degeneration [23]. Levels of neurofilaments, both light and heavy chains, have been shown to markedly increase in ALS, primarily due to rapid deterioration of motor neurons, and remain relatively stable during disease progression [24, 25]. Beyond its diagnostic potential, neurofilament light chain (NfL) has been found to correlate strongly with the progression rate and survival in ALS [26]. However, little research has been done to understand the correlations between clinical chemistry biomarkers and neurofilaments.

In this study, using a cohort of newly diagnosed patients with ALS in Stockholm, Sweden, we aimed to (1) assess the relationship between various biomarkers commonly measured in clinical chemistry and survival in ALS; (2) investigate the synergistic associations of multiple biomarkers with survival, longitudinal functional decline, and level of NfL measured in cerebrospinal fluid (CSF); and (3) assess if biomarkers measured though clinical chemistry provide additional value to existing prognostic factors.

## Methods

### Study design

We leveraged data from the ALSrisc Study (“Biomarkers and Risk Factors for Amyotrophic Lateral Sclerosis”), a case–control study including around 80% of newly diagnosed patients with ALS in the greater Stockholm area since 2016 onward [27]. The ALSrisc Study had a pilot phase in 2015, including patients diagnosed during 2014–2015. The present study included all patients with ALS included in the ALSrisc Study who had at least one measurement of clinical chemistry within 90 days before or 90 days after their reported date of diagnosis (i.e., “baseline”), leading to a total of 270 patients. The study period was from April 8th 2014 to May 18th 2021. We obtained clinical chemistry data from medical records among these patients, including biomarkers assessed in either blood (plasma, serum, or whole blood) or CSF. We excluded biomarkers with a high rate of missing values (> 25%), resulting in a set of 29 biomarkers for final analysis. These biomarkers could be classified into five categories: electrolytes, immunological biomarkers, neurological biomarkers, metabolic biomarkers, and hematopoietic biomarkers (Supplementary Table 1). The 25% cutoff for missing data was decided after considering the reliability of techniques dealing with missing values, particularly in the context of a relatively small sample size for a rare and heterogeneous disease like ALS, for which missingness patterns might be less predictable.

Through the cross-linkage between this study and the Swedish Motor Neuron Disease (MND) Quality Registry [28] using unique Swedish personal identity numbers, we obtained information on the demographic and clinical characteristics of all patients. A full list of the extracted variables can be found in Supplementary Table 2. We followed these patients from the date of diagnosis, until death, initiation of invasive ventilation, withdrawal from the study, or 13th of August 2022, whichever came first. The primary outcome during follow-up was either death or initiation of invasive ventilation, assessed at 6 months, 1 year, or 3 years post-diagnosis. In addition, we used a joint latent class model to combine changes in ALS functional rating scale-revised (ALSFRS-R) score and mortality data as a secondary outcome [29].

## Statistical analyses

We first summarized the characteristics of the study participants and compared these characteristics to that of all ALS patients diagnosed during the same period in Stockholm, according to the Swedish MND Quality Registry, to understand the representativeness of the study sample to its source population.

### Multiple imputation

The degree of missingness in the studied biomarkers varied from 2.6% to 24.9%. We employed the method of multiple imputation, using the Multiple Imputation by Chained Equations (MICE) package in R and the Classification and Regression Tree (CART) method, as it is shown to be robust against outliers, skewness, and multicollinearity and accommodate non-linear relationships [30, 31]. All variables (biomarkers, demographic and clinical characteristics) were used as predictors for the imputation process. Based on the average degree of missingness, we concluded that 20 datasets and 5 iterations were sufficient [32]. To assess the quality of the multiple imputed data sets, we plotted the distributions of the biomarkers in each imputed data set against the distributions of the original data set (Supplementary Fig. 1). To deal with non-normal distributions and skewness, we used the Adaptive Box–Cox transformation method to find the best transformation method for each biomarker, based on their distributions and with the utilization of the λ value [33]. After normalization, the biomarkers were standardized.

### Analysis 1

Biomarkers were treated as continuous variables and we used Cox proportional hazards model to assess the association of a 1-standard deviation (SD) increase in each biomarker measured at baseline with the risk of death or invasive ventilation use during 6 months, 1 year, or 3 years after ALS diagnosis, using time since diagnosis as the underlying time scale. We used both univariable and multivariable models. For the multivariable model, we controlled for the following variables: sex (male vs female), age at diagnosis (treated as a continuous variable), site of onset (bulbar vs non-bulbar), diagnostic delay (continuous variable), BMI at diagnosis (continuous variable), and the ALSFRS-R score at diagnosis (continuous variable). Diagnostic delay was defined as the time from symptom onset to diagnosis. Results derived from multiple imputed datasets were pooled using Rubin’s rules [34].

### Analysis 2

To investigate a possible synergistic effect of the biomarkers, we first conducted exploratory factor analysis (EFA), based on the data with multiple imputation, using the mifa package in R [35]. We calculated the average covariance matrix from all the separate covariance matrices among the different imputed data sets. We used the average covariance matrix to calculate the factor loadings. To assess the adequacy of our data for the factor analysis, we used the Kaiser–Meyer–Olkin (KMO) criterion and variables that had communalities less than 0.5 were excluded from this analysis. We used the maximum likelihood method to determine the efficient number of factors, demonstrated in scree plot and parallel analysis plot. We performed both orthogonal rotation, which assumes that the factors are uncorrelated, and oblique rotation, which allows the factors to be correlated, and the observed patterns were similar in both scenarios. For simplicity, we used the orthogonal rotation (varimax). We then used Cox proportional hazards model to assess the association between 1-SD increase of the identified factors and risk of death or invasive ventilation use during the 6 months, 1 year, or 3 years after ALS diagnosis. As the factors were not correlated, we included them all in the same model, initially without any adjustment for covariates and later with adjustment for the covariates mentioned in Analysis 1. The latter analysis was performed to assess the potential value of using the factors as prognostic indicators in ALS, independently of other known prognostic indicators. In a sensitivity analysis, we additionally adjusted for several other factors potentially related to prognosis, including presence of ALS gene (yes, no, or unknown), presence of dementia (yes or no), and riluzole use at the time of measurement of clinical chemistry markers (yes or no). Finally, we used multiple linear regression to assess the correlation between the identified factors and NfL levels measured in CSF at baseline, with or without multivariable adjustment. The linearity assumption was assessed based on the plotting of residuals.

### Analysis 3

We performed an analysis to examine whether the EFA-derived factors based on clinical chemistry markers could be predictive of trajectories of functional decline by time since ALS diagnosis. We first used a joint latent class model to identify distinct latent classes of functional decline trajectories including 242 of the 270 ALS patients who had at least 2 ALSFRS-R measurements. This model comprises three sub-models: (1) a multinomial logistic regression model to identify latent classes, (2) a linear mixed model to assess the temporal trend of ALSFRS-R, and (3) a survival model for time to death [29]. This integrated approach aims to minimize bias from informative censoring, e.g., due to death during follow-up, by combining longitudinal measures of functional status with survival data. The multinomial model, which determines latent classes, was not adjusted for any covariate. The linear mixed model was adjusted for sex, age at diagnosis, onset site, and time since diagnosis, whereas the survival model was adjusted for sex, age at diagnosis, and onset site. Patients were allocated to the class for which they had the highest probability of belonging. The number of classes was determined based on the Bayesian Information Criterion (BIC) and clinical meaningfulness. We then used multinomial logistic regression model to assess the associations of the EFA-derived factors and NfL in CSF with the membership in different latent classes. Factors were added to the same model with or without multivariable adjustment. To assess the robustness of the results, in a sensitivity analysis, we excluded from the analysis the patients who had a probability of ≤ 70% in belonging to a specific latent class.

All statistical analyses were conducted in R software (R 4.3.1). A two-sided *p < *0.05 was considered statistically significant.

## Results

The mean age of diagnosis was 66 years and the ratio between men and women was almost 1:1 among the study participants (Table 1). Approximately one third of the patients had bulbar onset. By the end of the study, 213 (78.9%) patients had died or initiated the use of invasive ventilation. Patients diagnosed with ALS in Stockholm during the same period who were not included in the study were slightly older, compared to the patients included in the present study, at the time of diagnosis. Otherwise, there was no substantial difference between the two groups.

**Table 1:** Baseline characteristics of patients with ALS included in the study

Characteristics	Patients included in the study (*N* = 270)	Patients diagnosed in Stockholm during the same period who were not part of the study (*N* = 132)	*p*^a^
Age at diagnosis, years			0.05
Mean (SD)	65.9 (11.1)	68.4 (12.3)	
Sex, *N* %			0.50
Male	136 (50.4%)	61 (46.2%)	
Female	134 (49.6%)	71 (53.8%)	
Onset site, *N* %			0.27
Non-bulbar	171 (63.3%)	86 (65.2%)	
Bulbar	98 (36.9%)	37 (28%)	
Unknown	1 (0.4%)	9 (6.8%)	
Diagnostic delay, months			0.83
Median (IQR)	12.57 (10.55)	12.23 (14.15)	
Death or initiation of invasive ventilation			0.14
*N* (%)	213 (78.9%)	113 (85.6%)	

^a^Derived from Student’s *t* test, Chi-square test, or Mood’s median test

### Analysis 1

**Fig. 1:**
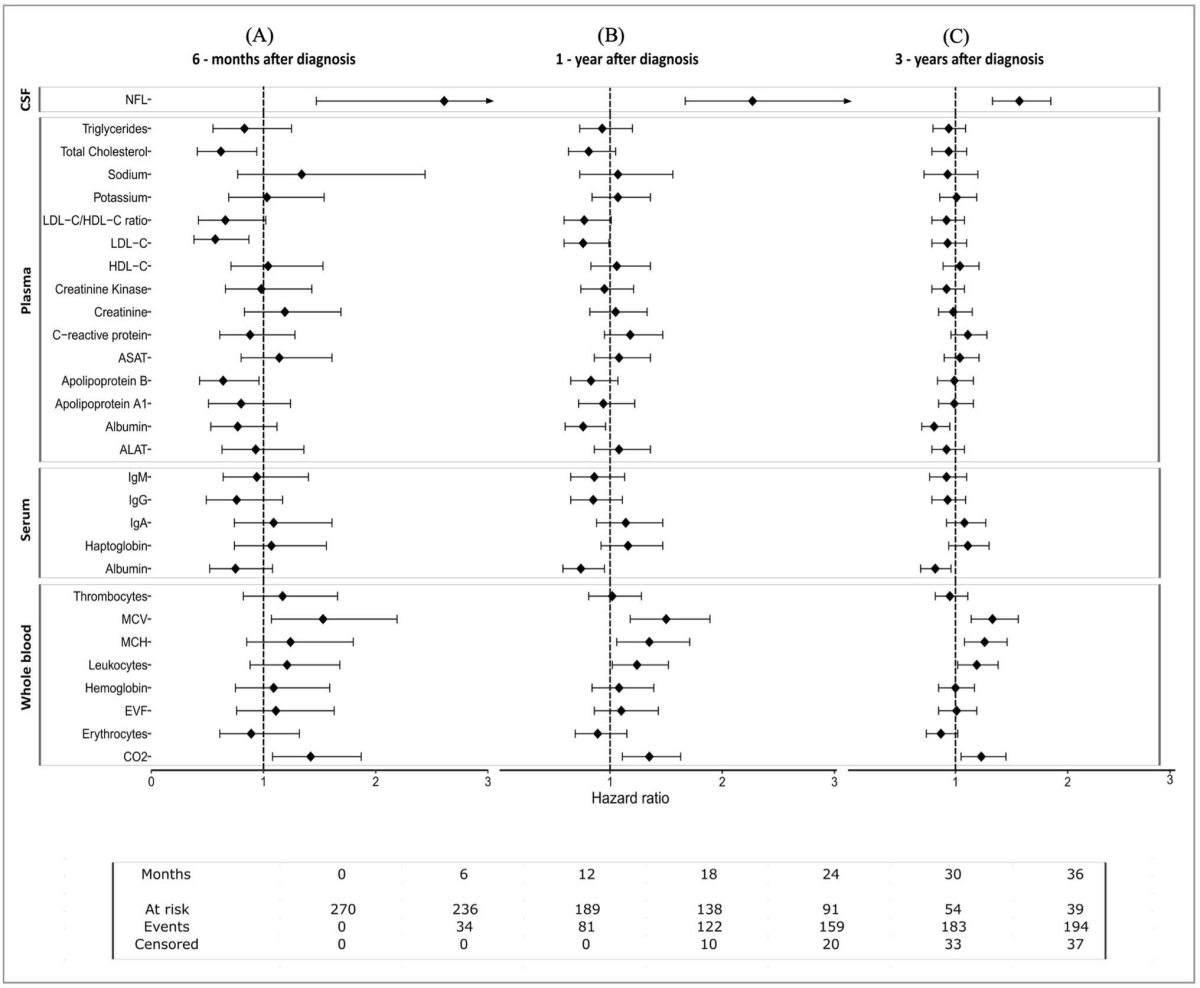
Associations of clinical chemistry biomarkers (per 1-SD increase) with risk of mortality after ALS diagnosis (6 months, 1 year, and 3 years since diagnosis), using Cox proportional hazard models after multiple imputation, normalization, and standardization

During the 6 months after ALS diagnosis, several blood biomarkers exhibited a significant association with the risk of death (or the use of invasive ventilation), including total cholesterol (HR: 0.62, 95%CI 0.41–0.94), low-density lipoprotein (LDL-C, HR: 0.57, 95%CI 0.38–0.87), apolipoprotein B (HR: 0.64, 95%CI 0.43–0.96), mean corpuscular volume (MCV, HR: 1.53, 95%CI 1.07–2.19), and carbon dioxide (CO_2_, HR: 1.42, 95%CI 1.08–1.87), in addition to NfL in CSF (HR: 2.61, 95%CI 1.47–4.65) (Fig. 1A). During the 1 year after diagnosis, LDL-C (HR: 0.76, 95%CI 0.59–0.99), serum albumin (HR: 0.74, 95%CI 0.58–0.95), and plasma albumin (HR: 0.76, 95%CI 0.60–0.96) were associated with a lower risk of death, whereas count of leukocytes (HR: 1.24, 95%CI 1.02–1.52), mean corpuscular hemoglobin (MCH, HR: 1.35, 95%CI 1.06–1.71), MCV (HR: 1.50, 95%CI 1.18–1.89), CO_2_ (HR: 1.35, 95%CI 1.11–1.63), and NfL (HR: 2.27, 95%CI 1.67–3.10) were associated with an increased risk of death (Fig. 1B). During the 3 years after diagnosis, albumin in serum or plasma was still associated with a lower risk of death, whereas MCV, MCH, leukocytes count, CO_2_, and NfL were still associated with a higher risk of death (Fig. 1C).

After multivariable adjustment, hemoglobin was associated with a higher risk of death at all time points, although the effect size declined with increasing time since diagnosis [6 months (HR: 2.07, 95%CI 1.28–3.37), 1 year (HR: 1.45, 95%CI 1.06–1.97), and 3 years (HR: 1.20, 95%CI 0.99–1.46)] (Supplementary Table 3). Erythrocyte volume fraction (EVF) exhibited a similar trend [6 months (HR: 1.70, 95%CI 1.09–2.65), 1 year (HR: 1.39, 95%CI 1.03–1.88), and 3 years (HR: 1.18, 95%CI 0.97–1.42)]. In addition, erythrocytes count was associated with a higher risk of death during the 6 months after diagnosis (HR: 1.53 95%CI 1.01–2.32), although not thereafter.

### Analysis 2

**Fig. 2:**
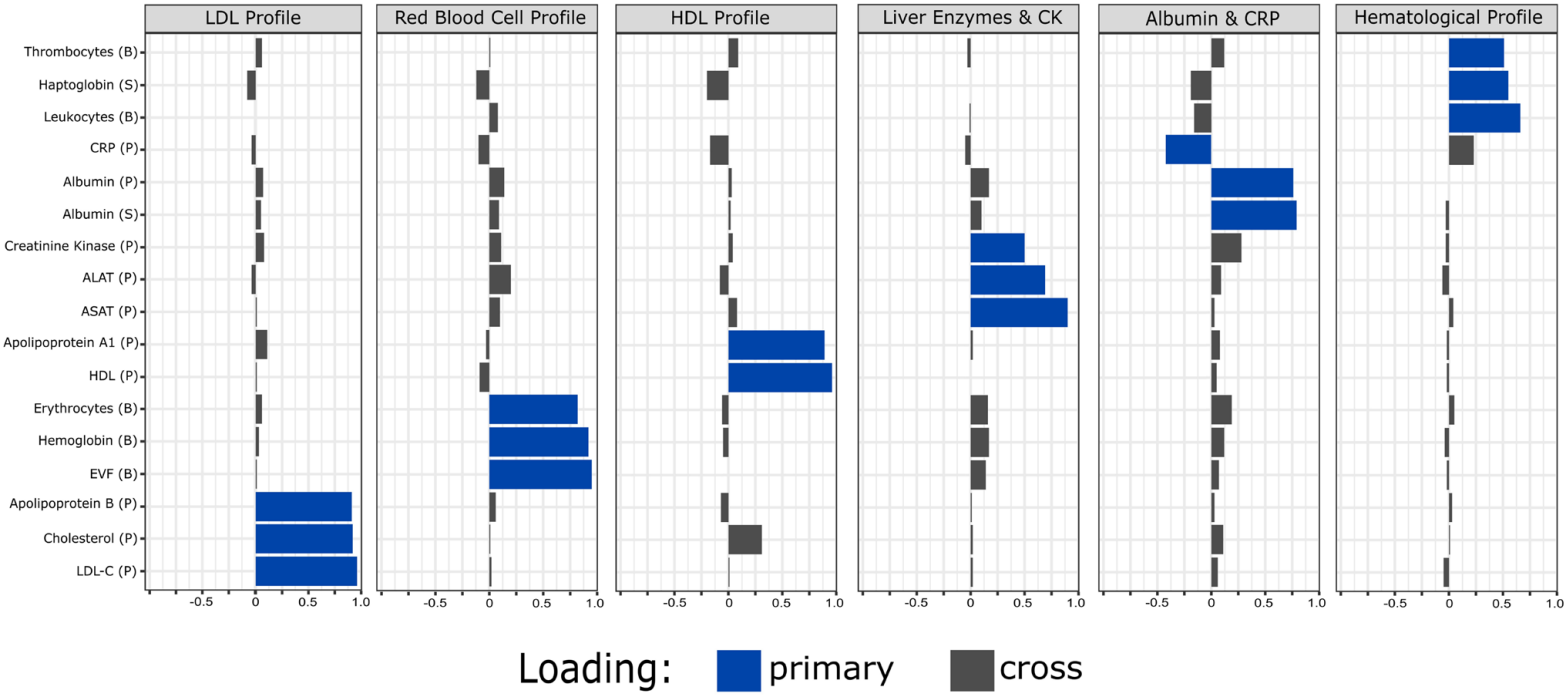
Exploratory factor analysis showing the six factors that explained 68% of the total variance in the clinical chemistry data, with blue color indicating the primary variable loadings included in each factor**.** Weights were calculated using the absolute value of the loadings

In EFA, six uncorrelated factors were identified among the clinical chemistry biomarkers studied, based on their factor loadings (Fig. 2).

These factors were named according to their predominant loaded variables. The “LDL profile” factor was associated with a lower risk of death during 6 months after ALS diagnosis, while the “albumin & CRP” factor was associated with a lower risk of death during 1 year or 3 years after ALS diagnosis (Table 2).

**Table 2:** Associations of EFA-derived factors (per 1-SD increase) with risk of mortality after ALS diagnosis (6 months, 1 year, and 3 years after diagnosis) or baseline level of neurofilament light chain (NfL) in CSF

Factor	6 months after diagnosis^i^ (*N *= 270)					1 year after diagnosis^i^ (*N* = 270)			
	HR (95% CI) Unadjusted^a^		HR (95% CI) Adjusted^b^			HR (95% CI) Unadjusted^a^			HR (95% CI) Adjusted^b^
LDL profile	0.58 (0.38–0.88)		0.84 (0.55–1.28)			0.76 (0.59–0.99)			0.96 (0.75–1.23)
Red blood cell profile	1.16 (0.82–1.65)		1.65 (1.07–2.65)			1.20 (0.94–1.54)			1.37 (1.03–1.83)
HDL profile	1.07 (0.74–1.55)		1.14 (0.72–1.80)			1.10 (0.87–1.40)			1.21 (0.92–1.59)
Liver enzymes and CK	1.05 (0.75–1.49)		1.24 (0.85–1.80)			1.04 (0.84–1.30)			1.11 (0.88–1.39)
Albumin and CRP	0.78 (0.55–1.10)		1.27 (0.84–1.93)			0.70 (0.56–0.88)			0.94 (0.72–1.22)
Hematological profile	1.12 (0.81–1.55)		0.99 (0.70–1.38)			1.13 (0.93–1.37)			1.08 (0.88–1.32)

Factor		3 years after diagnosis^i^ (*N* = 270)					Baseline NfL (CSF)^ii^ (*N* = 270)		
		HR (95% CI) Unadjusted^a^		HR (95% CI) Adjusted^b^			β (95% CI) Unadjusted^a^	β (95% CI) Adjusted^b^	
LDL profile		0.91 (0.90–1.24)		1.03 (0.86–1.22)			0.02 (−0.11 ~ 0.16)	0.07 (−0.06–0.19)	
Red blood cell profile		1.06 (0.90–1.24)		1.16 (0.97–1.39)			0.001 (−0.13 ~ 0.13)	−0.01 (−0.14–0.12)	
HDL profile		1.04 (0.89–1.21)		1.05 (0.88–1.25)			−0.03 (−0.17 ~ 0.11)	0.03 (−0.11–0.18)	
Liver enzymes and CK		0.96 (0.83–1.10)		1.05 (0.91–1.21)			0.12 (−0.02 ~ 0.25)	0.08 (−0.06–0.22)	
Albumin and CRP		0.77 (0.66–0.89)		0.96 (0.80–1.14)			–0.01 (−0.13 ~ 0.12)	0.04 (−0.09–0.17)	
Hematological profile		1.09 (0.95–1.24)		1.03 (0.89–1.19)			0.05 (−0.08 ~ 0.18)	0.02 (−0.11–0.15)	

^i^Derived from multivariable Cox proportional hazards model

^ii^Derived from multiple linear regression

^a^Unadjusted for other covariates

^b^Adjusted for sex, age at diagnosis, onset site, ALSFRS-R score at diagnosis, BMI at diagnosis, and diagnostic delay

While these associations diminished after multivariable adjustment, the ‘Red blood cell profile’ factor was still associated with an increased mortality risk at 6 months and 1 year post-diagnosis. In the sensitivity analysis with additional adjustment for genetic mutation, dementia, and riluzole use, 37 patients were found to have a genetic mutation, 23 to have dementia at the time of diagnosis, and 150 to be using riluzole. This additional adjustment did not lead to largely changed results (Supplementary Table 4). Finally, although none of the factors reached statistically significant results in relation to NfL levels in CSF, there was a notable trend for the “Liver enzymes & CK” factor in both models.

### Analysis 3

**Fig. 3:**
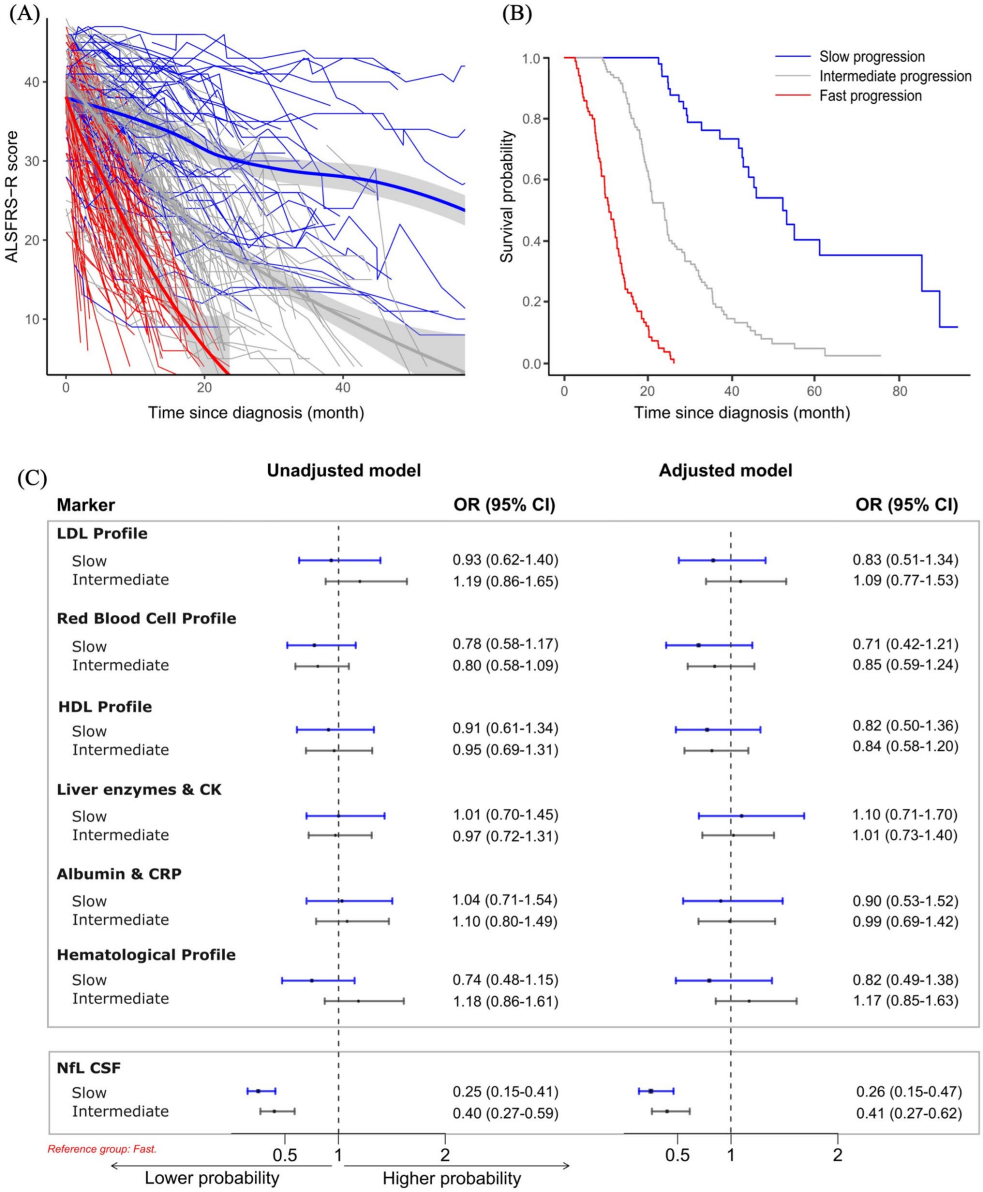
**A** Spaghetti plot showing three trajectories of ALSFRS-R score by time since ALS diagnosis. Derived from joint trajectory model for latent classes. Linear regression model was adjusted for sex, age at diagnosis, onset site, and time since diagnosis. Survival model was adjusted for sex, age at diagnosis, and onset site. **B** Survival curves for patients with the three trajectories of ALSFRS-R score by time since ALS diagnosis. **C** Forest plot displaying odds ratio (OR) and 95% confidence interval (CI) of belonging to different trajectories of ALSFRS-R score in relation to the six factors and neurofilament light chain (NfL) in CSF, derived from multinomial logistic regression. All factors were included in the same multivariable model, after adjustment for sex, age at diagnosis, onset site, ALSFRS-R score at diagnosis, BMI at diagnosis, and diagnostic delay

For the joint model, we selected the model with three trajectory classes, showing distinct trajectories of ALSFRS-R decline over time (Fig. 3A). Specifically, 49 patients were assigned to the slow, 107 to the intermediate, and 86 to the fast progression classes. The survival plot of the three classes presents consistent trend as ALSFRS-R decline (Fig. 3B). Although the associations between the six factors and trajectory classes did not reach statistical significance, patients with higher values in the 'Red blood cell profile' factor were less likely to be classified into the slow or intermediate classes, regardless of multivariable adjustment (Fig. 3C). NfL in CSF showed significant association with class membership. Specifically, patients with higher level of NfL were less likely to be classified into slow or intermediate classes, compared to fast progression class. When we changed the probability threshold for class membership to 70%, we did not notice significant change in the results (Supplementary Tables 5 and 6).

## Discussion

In this study, we performed a comprehensive analysis using clinical chemistry data from 270 patients with ALS to examine their association with survival and disease progression, individually and collectively. We observed that increased levels of total cholesterol, LDL-C, apolipoprotein B, and albumin at diagnosis were protective factors for survival. However, the strength of the associations diminished over time and, among these biomarkers, only albumin was associated with survival throughout the 3 years post-diagnosis. Conversely, increased levels of blood leukocyte counts, MCV, MCH, CO_2_, and NfL in CSF were associated with a higher risk of mortality during all the 3 years after diagnosis. In factor analysis, we identified six uncorrelated factors among the studied clinical chemistry biomarkers, among which the composite ‘LDL profile’ and ‘albumin & CRP’ factors were associated with a decreased risk of mortality after ALS diagnosis. After adjusting for other prognostic indicators, however, only the ‘Red blood cell profile’ factor was associated with increased risk of mortality across 3 years after ALS diagnosis. Interestingly, we did not observe an association between the ‘Red blood cell profile’ factor and NfL level in CSF measured at the time of diagnosis.

Our results of individual biomarkers are generally consistent with prior research on albumin, lipids, apolipoproteins, and neurofilaments [14, 16, 36–38]. We showed further that the predictive value of these biomarkers declined over time in general, and that some biomarkers were predictive of survival only during the first year (e.g., total cholesterol, LDL-C, and apolipoprotein B), whereas others during the entire 3 years (e.g., albumin, MCV, CO_2_, and NfL) after ALS diagnosis. Albumin, for instance, is associated with a lower risk of death throughout the 3 years after ALS diagnosis. Albumin regulates plasma oncotic pressure and transports physiological metabolites [39] and lower levels of blood albumin (hypoalbuminemia) have been associated with poor nutrition or inflammation [40, 41]. This finding supports further the importance of nutritional support and inflammation control for patients with ALS [21, 42]. In addition, we found that elevated CO_2_ levels in venous blood served as a prognostic indicator in ALS, both in the short (within 6 months) and long (within 3 years) run. Elevated CO_2_ levels in blood can indicate impaired respiratory function and hypercapnia, which are common complications in ALS due to respiratory muscles atrophy and insufficient removal of CO_2_ [43, 44]. Similarly, serum chloride, which plays a critical role in maintaining hydration, muscle function, and pH balance, has been found to be associated with respiratory failure and overall survival in patients with ALS [45]. Hypercapnia is one of the indication criteria for the initiation of non-invasive ventilation (NIV) [46, 47]. As shown in a randomized clinical trial, early intervention with NIV slows the decline of respiratory function and minimizes complications [48]. Therefore, early assessment of the presence of respiratory failure or hypercapnia is essential in the management of patients with ALS.

Another interesting finding in this study pertains to red blood cells. MCV, which measures average volume of a red blood cell, demonstrated predictive ability during the entire 3 years after ALS diagnosis. In the analysis after adjustment for known prognostic indicators of ALS, hemoglobin, EVF, and count of erythrocytes, which are major components of the ‘Red blood cell profile’ factor, were all associated with risk of death after ALS diagnosis, highlighting the potential utility of these biomarkers as additional biomarkers in the prognostic prediction of ALS patients. To the best of our knowledge, only one previous study has examined hemoglobin in relation to ALS survival, showing that an increased level of hemoglobin was associated with increased odds of death in ALS patients [38]. The lack of correlation between the ‘Red blood cell profile’ factor and CSF NfL at diagnosis suggests that the ‘Red blood cell profile’ factor may not be indicative of the severity of neurodegeneration in ALS but rather supports a role of the bodily systems outside of the central nervous system. For instance, a higher level of chronic hypoxia in ALS patients might lead to a higher level of red blood cells, as a compensating mechanism [49]. Similarly, as hemoglobin is a transporter of oxygen [50], a higher level of hemoglobin can indicate a higher level of oxidative stress [51]. Oxidative stress is caused by an overabundance of reactive oxygen species (ROS) or a failure in the antioxidant defense system, leading to various changes like alteration of the redox state of vital proteins and their function [52].

NfL is a known biomarker for ALS [53, 54]. In line with this, the present study found a significant association of NfL measured in CSF at diagnosis with both mortality and functional decline after ALS diagnosis. The ‘Liver enzyme & CK’ factor showed a positive association with NfL at baseline, both before and after multivariable adjustment, although the results were not statistically significant. Research in mice has indicated that liver damage may play a role in the progression of ALS by influencing the liver’s production of proteins linked to inflammation and oxidative stress as well as proteins that cause liver fibrosis [55]. However, future research is required to validate this finding.

The strength of our study lies in the comprehensive approach to examine the association of a diverse array of clinical chemistry biomarkers, both individually and jointly, with survival outcomes in different time windows after ALS diagnosis, as well as with functional decline and NfL measured in CSF. Another strength is the complete follow-up of all patients, as well as the combined application of EFA and Cox model, which mitigated potential multicollinearity and allowed the assessment of multiple factors within the same model without inflating Type 1 error. Our study also has limitations. The wide confidence intervals in some of the results suggest that we might have failed to identify associations with small effect sizes, due to limited statistical power, in some of the analyses. The number of measurements of biomarkers was not identical among patients with ALS. Although multiple imputation was applied under the assumption that data were missing at random, which is less stringent than the assumption of missing completely at random in complete case analysis, patients with different disease severities might have received different number of biomarker measurements. Further, given the exploratory nature of Analysis 1 and the high correlations between many of the clinical chemistry markers, we did not apply correction of multiple testing in Analysis 1. Subsequently, the results of this analysis should be interpreted with caution. Finally, although findings of this study are likely generalizable to all ALS patients in Stockholm, given the similar clinical characteristics noted between the study sample and its source population (i.e., all ALS patients diagnosed during the study period in Stockholm), validation from future studies with ALS patients of other areas is needed.

## Conclusion

Our study showed the potential of routine clinical chemistry markers in predicting survival and prognosis of ALS. Several biomarkers, including lipids, albumin, leukocytes count, MCV, MCH, and CO_2_, were associated with patient survival. The ‘Red blood cell profile’ factor showed additional prognostic value on top of other established prognostic indicators. As these blood markers provide insights into bodily functions outside of the central nervous system, integrating them in the care of ALS patients might lead to a more holistic management strategy for the disease.

## Supplementary Information

Below is the link to the electronic supplementary material.

Supplementary file1 (DOCX 2778 KB)

## Data Availability

Data used in the present study are not possible to share publicly due to Swedish and European regulations. Please contact the corresponding author for more information.

## References

[1] Brown RH, Al-Chalabi A (2017) Amyotrophic lateral sclerosis. N Engl J Med 377(2):162–172 28700839 10.1056/NEJMra1603471

[2] Pinto S, Gromicho M, Oliveira Santos MO, Swash M, De Carvalho M (2023) Respiratory onset in amyotrophic lateral sclerosis: clinical features and spreading pattern. Amyotroph Lateral Scler Frontotemporal Degener 24(1–2):40–44 35510537 10.1080/21678421.2022.2067777

[3] Strong MJ, Abrahams S, Goldstein LH, Woolley S, Mclaughlin P, Snowden J et al (2017) Amyotrophic lateral sclerosis—frontotemporal spectrum disorder (ALS-FTSD): revised diagnostic criteria. Amyotroph Lateral Scler Frontotemporal Degener 18(3–4):153–174 28054827 10.1080/21678421.2016.1267768 PMC7409990

[4] Bersano E, Sarnelli MF, Solara V, Iazzolino B, Peotta L, De Marchi F et al (2020) Decline of cognitive and behavioral functions in amyotrophic lateral sclerosis: a longitudinal study. Amyotroph Lateral Scler Frontotemporal Degener 21(5–6):373–379 32484726 10.1080/21678421.2020.1771732

[5] Chiò A, Moglia C, Canosa A, Manera U, Vasta R, Brunetti M et al (2019) Cognitive impairment across ALS clinical stages in a population-based cohort. Neurology 93(10):e984 31409738 10.1212/WNL.0000000000008063 PMC6745732

[6] Mulder D, Kurland L, Offord K, Beard C (1986) Familial adult motor neuron disease: amyotrophic lateral sclerosis. Neurology 36(4):511–517 3960325 10.1212/wnl.36.4.511

[7] Sennfält S, Kläppe U, Thams S, Samuelsson K, Press R, Fang F et al (2023) The path to diagnosis in ALS: delay, referrals, alternate diagnoses, and clinical progression. Amyotroph Lateral Scler Frontotemporal Degener 24(1–2):45–53 35343340 10.1080/21678421.2022.2053722

[8] Gwathmey KG, Corcia P, McDermott CJ, Genge A, Sennfält S, de Carvalho M et al (2023) Diagnostic delay in amyotrophic lateral sclerosis. Eur J Neurol 30(9):2595–2601 37209406 10.1111/ene.15874

[9] Masrori P, Van Damme P (2020) Amyotrophic lateral sclerosis: a clinical review. Eur J Neurol 27(10):1918–1929 32526057 10.1111/ene.14393 PMC7540334

[10] Chiò A, Logroscino G, Hardiman O, Swingler R, Mitchell D, Beghi E et al (2009) Prognostic factors in ALS: a critical review. Amyotroph Lateral Scler 10(5–6):310–323 19922118 10.3109/17482960802566824 PMC3515205

[11] Al-Chalabi A, Hardiman O (2013) The epidemiology of ALS: a conspiracy of genes, environment and time. Nat Rev Neurol 9(11):617–628 24126629 10.1038/nrneurol.2013.203

[12] Westeneng HJ, Debray TPA, Visser AE, van Eijk RPA, Rooney JPK, Calvo A et al (2018) Prognosis for patients with amyotrophic lateral sclerosis: development and validation of a personalised prediction model. Lancet Neurol 17(5):423–433 29598923 10.1016/S1474-4422(18)30089-9

[13] Ahmad A, Imran M, Ahsan H (2023) Biomarkers as biomedical bioindicators: approaches and techniques for the detection, analysis, and validation of novel biomarkers of diseases. Pharmaceutics 15(6):1630 37376078 10.3390/pharmaceutics15061630 PMC10303887

[14] Ingre C, Chen L, Zhan Y, Termorshuizen J, Yin L, Fang F (2020) Lipids, apolipoproteins, and prognosis of amyotrophic lateral sclerosis. Neurology 94(17):e1835–e1844 32221024 10.1212/WNL.0000000000009322 PMC7274849

[15] Michels S, Kurz D, Rosenbohm A, Peter RS, Just S, Bäzner H et al (2023) Association of blood lipids with onset and prognosis of amyotrophic lateral sclerosis: results from the ALS Swabia registry. J Neurol 270(6):3082–3090 36853389 10.1007/s00415-023-11630-4 PMC10193299

[16] Chiò A, Calvo A, Bovio G, Canosa A, Bertuzzo D, Galmozzi F et al (2014) Amyotrophic lateral sclerosis outcome measures and the role of albumin and creatinine. JAMA Neurol 71(9):1134 25048026 10.1001/jamaneurol.2014.1129

[17] Guo Q, Hu W, Xu L, Luo H, Wang N, Zhang Q (2021) Decreased serum creatinine levels predict short survival in amyotrophic lateral sclerosis. Ann Clin Transl Neurol 8(2):448–455 33449454 10.1002/acn3.51299 PMC7886033

[18] Kharel S, Ojha R, Preethish-Kumar V, Bhagat R (2022) C-reactive protein levels in patients with amyotrophic lateral sclerosis: a systematic review. Brain Behav 12(3):e2532 35201675 10.1002/brb3.2532 PMC8933772

[19] Dupuis L, Pradat PF, Ludolph AC, Loeffler JP (2011) Energy metabolism in amyotrophic lateral sclerosis. Lancet Neurol 10(1):75–82 21035400 10.1016/S1474-4422(10)70224-6

[20] Cunha-Oliveira T, Montezinho L, Mendes C, Firuzi O, Saso L, Oliveira PJ et al (2020) Oxidative stress in amyotrophic lateral sclerosis: pathophysiology and opportunities for pharmacological intervention. Oxid Med Cell Longev 15(2020):1–29 10.1155/2020/5021694 PMC7683149 33274002

[21] Ludolph AC, Dorst J, Dreyhaupt J, Weishaupt JH, Kassubek J, Weiland U et al (2020) Effect of high-caloric nutrition on survival in amyotrophic lateral sclerosis. Ann Neurol 87(2):206–216 31849093 10.1002/ana.25661

[22] Chico L, Ienco EC, Bisordi C, Lo Gerfo A, Petrozzi L, Petrucci A et al (2018) Amyotrophic lateral sclerosis and oxidative stress: a double-blind therapeutic trial after curcumin supplementation. CNS Neurol Disord Drug Targets 17(10):767–779 30033879 10.2174/1871527317666180720162029

[23] Witzel S, Mayer K, Oeckl P (2022) Biomarkers for amyotrophic lateral sclerosis. Curr Opin Neurol 35(5):699–704 35942674 10.1097/WCO.0000000000001094

[24] Rosengren LE, Karlsson J, Karlsson J, Persson LI, Wikkelsø C (1996) Patients with amyotrophic lateral sclerosis and other neurodegenerative diseases have increased levels of neurofilament protein in CSF. J Neurochem 67(5):2013–2018 8863508 10.1046/j.1471-4159.1996.67052013.x

[25] Brettschneider J, Petzold A, Süßmuth SD, Ludolph AC, Tumani H (2006) Axonal damage markers in cerebrospinal fluid are increased in ALS. Neurology 66(6):852–856 16567701 10.1212/01.wnl.0000203120.85850.54

[26] Benatar M, Zhang L, Wang L, Granit V, Statland J, Barohn R et al (2020) Validation of serum neurofilaments as prognostic and potential pharmacodynamic biomarkers for ALS. Neurology 95(1):59 10.1212/WNL.0000000000009559 PMC7371380 32385188

[27] Chourpiliadis C, Seitz C, Lovik A, Joyce EE, Pan L, Hu Y et al (2024) Lifestyle and medical conditions in relation to ALS risk and progression—an introduction to the Swedish ALSrisc Study. J Neurol 271(8):5447–5459 38878106 10.1007/s00415-024-12496-w PMC11319377

[28] Longinetti E, Regodón Wallin A, Samuelsson K, Press R, Zachau A, Ronnevi LO et al (2018) The Swedish motor neuron disease quality registry. Amyotroph Lateral Scler Frontotemporal Degener 19(7–8):528–537 30296856 10.1080/21678421.2018.1497065

[29] Kyheng M, Babykina G, Ternynck C, Devos D, Labreuche J, Duhamel A (2021) Joint latent class model: simulation study of model properties and application to amyotrophic lateral sclerosis disease. BMC Med Res Methodol 21(1):198 34592944 10.1186/s12874-021-01377-9 PMC8482570

[30] Breiman L, Friedman JH, Olshen RA, Stone CJ (2017) Classification and regression trees. Routledge, Boca Raton

[31] Austin PC, White IR, Lee DS, van Buuren S (2021) Missing data in clinical research: a tutorial on multiple imputation. Can J Cardiol 37(9):1322–1331 33276049 10.1016/j.cjca.2020.11.010 PMC8499698

[32] White IR, Royston P, Wood AM (2011) Multiple imputation using chained equations: Issues and guidance for practice. Stat Med 30(4):377–399 21225900 10.1002/sim.4067

[33] Yu H, Sang P, Huan T (2022) Adaptive box-cox transformation: a highly flexible feature-specific data transformation to improve metabolomic data normality for better statistical analysis. Anal Chem 94(23):8267–8276 35657711 10.1021/acs.analchem.2c00503

[34] Rubin DB (2018) Multiple imputation. Flexible imputation of missing data, 2nd Edition. Chapman and Hall/CRC, pp 29–62

[35] Nassiri V, Lovik A, Molenberghs G, Verbeke G (2018) On using multiple imputation for exploratory factor analysis of incomplete data. Behav Res Methods 50(2):501–517 29392587 10.3758/s13428-017-1013-4

[36] Chiò A, Calvo A, Ilardi A, Cavallo E, Moglia C, Mutani R et al (2009) Lower serum lipid levels are related to respiratory impairment in patients with ALS. Neurology 73(20):1681–1685 19917991 10.1212/WNL.0b013e3181c1df1e

[37] Gagliardi D, Meneri M, Saccomanno D, Bresolin N, Pietro CG, Corti S (2019) Diagnostic and prognostic role of blood and cerebrospinal fluid and blood neurofilaments in amyotrophic lateral sclerosis: a review of the literature. Int J Mol Sci 20(17):4152 31450699 10.3390/ijms20174152 PMC6747516

[38] Mandrioli J, Rosi E, Fini N, Fasano A, Raggi S, Fantuzzi AL et al (2017) Changes in routine laboratory tests and survival in amyotrophic lateral sclerosis. Neurol Sci 38(12):2177–2182 28980128 10.1007/s10072-017-3138-8

[39] Ha CE, Bhagavan NV (2013) Novel insights into the pleiotropic effects of human serum albumin in health and disease. Biochimica et Biophysica Acta (BBA) General Subjects 1830(12):5486–5493 23602811 10.1016/j.bbagen.2013.04.012

[40] Keller U (2019) Nutritional laboratory markers in malnutrition. J Clin Med 8(6):775 31159248 10.3390/jcm8060775 PMC6616535

[41] Don BR, Kaysen G (2004) Poor nutritional status and inflammation: serum albumin: relationship to inflammation and nutrition. Semin Dial 17(6):432–437 15660573 10.1111/j.0894-0959.2004.17603.x

[42] Khalid SI, Ampie L, Kelly R, Ladha SS, Dardis C (2017) Immune modulation in the treatment of amyotrophic lateral sclerosis: a review of clinical trials. Front Neurol 25:8 10.3389/fneur.2017.00486 PMC5622209 28993751

[43] Patel S, Miao JH, Yetiskul E, Anokhin A, Majmundar SH (2024) Physiology, carbon dioxide retention. In StatPearls. StatPearls Publishing 29494063

[44] Dorst J, Behrendt G, Ludolph AC (2019) Non-invasive ventilation and hypercapnia-associated symptoms in amyotrophic lateral sclerosis. Acta Neurol Scand 139(2):128–134 30394534 10.1111/ane.13043

[45] Manera U, Grassano M, Matteoni E, Bombaci A, Vasta R, Palumbo F et al (2023) Serum chloride as a respiratory failure marker in amyotrophic lateral sclerosis. Front Aging Neurosci 15:1188827 37293667 10.3389/fnagi.2023.1188827 PMC10244551

[46] Windisch W, Walterspacher S, Siemon K, Geiseler J, Sitter H (2010) Guidelines for non-invasive and invasive mechanical ventilation for treatment of chronic respiratory failure. Pneumologie 64(10):640–652 20799159 10.1055/s-0030-1255558

[47] Miller RG, Jackson CE, Kasarskis EJ, England JD, Forshew D, Johnston W et al (2009) Practice Parameter update: the care of the patient with amyotrophic lateral sclerosis: multidisciplinary care, symptom management, and cognitive/behavioral impairment (an evidence-based review). Neurology 73(15):1227–1233 19822873 10.1212/WNL.0b013e3181bc01a4 PMC2764728

[48] Sarasate M, González N, Córdoba-Izquierdo A, Prats E, Gonzalez-Moro JMR, Martí S et al (2023) Impact of early non-invasive ventilation in amyotrophic lateral sclerosis: a multicenter randomized controlled trial. J Neuromuscul Dis 10(4):627–637 37212068 10.3233/JND-221658 PMC10357175

[49] Webb KL, Dominelli PB, Baker SE, Klassen SA, Joyner MJ, Senefeld JW et al (2022) Influence of high hemoglobin-oxygen affinity on humans during hypoxia. Front Physiol 14:12 10.3389/fphys.2021.763933 PMC8795792 35095551

[50] Marengo-Rowe AJ (2006) Structure-function relations of human hemoglobins. Baylor Univ Med Center Proc 19(3):239–245 10.1080/08998280.2006.11928171 PMC1484532 17252042

[51] Reeder BJ, Wilson MT (2005) Hemoglobin and myoglobin associated oxidative stress: from molecular mechanisms to disease states. Curr Med Chem 12(23):2741–2751 16305469 10.2174/092986705774463021

[52] Barber SC, Mead RJ, Shaw PJ (2006) Oxidative stress in ALS: a mechanism of neurodegeneration and a therapeutic target. Biochimica et Biophysica Acta (BBA) Mol Basis Dis 1762(1112):1051–1067 10.1016/j.bbadis.2006.03.008 16713195

[53] Lu CH, Macdonald-Wallis C, Gray E, Pearce N, Petzold A, Norgren N et al (2015) Neurofilament light chain. Neurology 84(22):2247–2257 25934855 10.1212/WNL.0000000000001642 PMC4456658

[54] Su WM, Cheng YF, Jiang Z, Duan QQ, Yang TM, Shang HF et al (2021) Predictors of survival in patients with amyotrophic lateral sclerosis: a large meta-analysis. EBioMedicine 74:103732 34864363 10.1016/j.ebiom.2021.103732 PMC8646173

[55] Lee SH, Yang EJ (2018) Relationship between liver pathology and disease progression in a murine model of amyotrophic lateral sclerosis. Neurodegener Dis 18(4):200–207 30130789 10.1159/000491392

