# Supplementary material for: Exploring clinical chemistry markers in amyotrophic lateral sclerosis: insights into survival and disease trajectories
Source: J Neurol. 2024 Dec 12;272(1):7. doi: 10.1007/s00415-024-12774-7 (PMC11638388; doi:10.1007/s00415-024-12774-7)
Supplement: Supplementary file 1 — Supplementary file1 (DOCX 2778 KB) [file 415_2024_12774_MOESM1_ESM.docx]

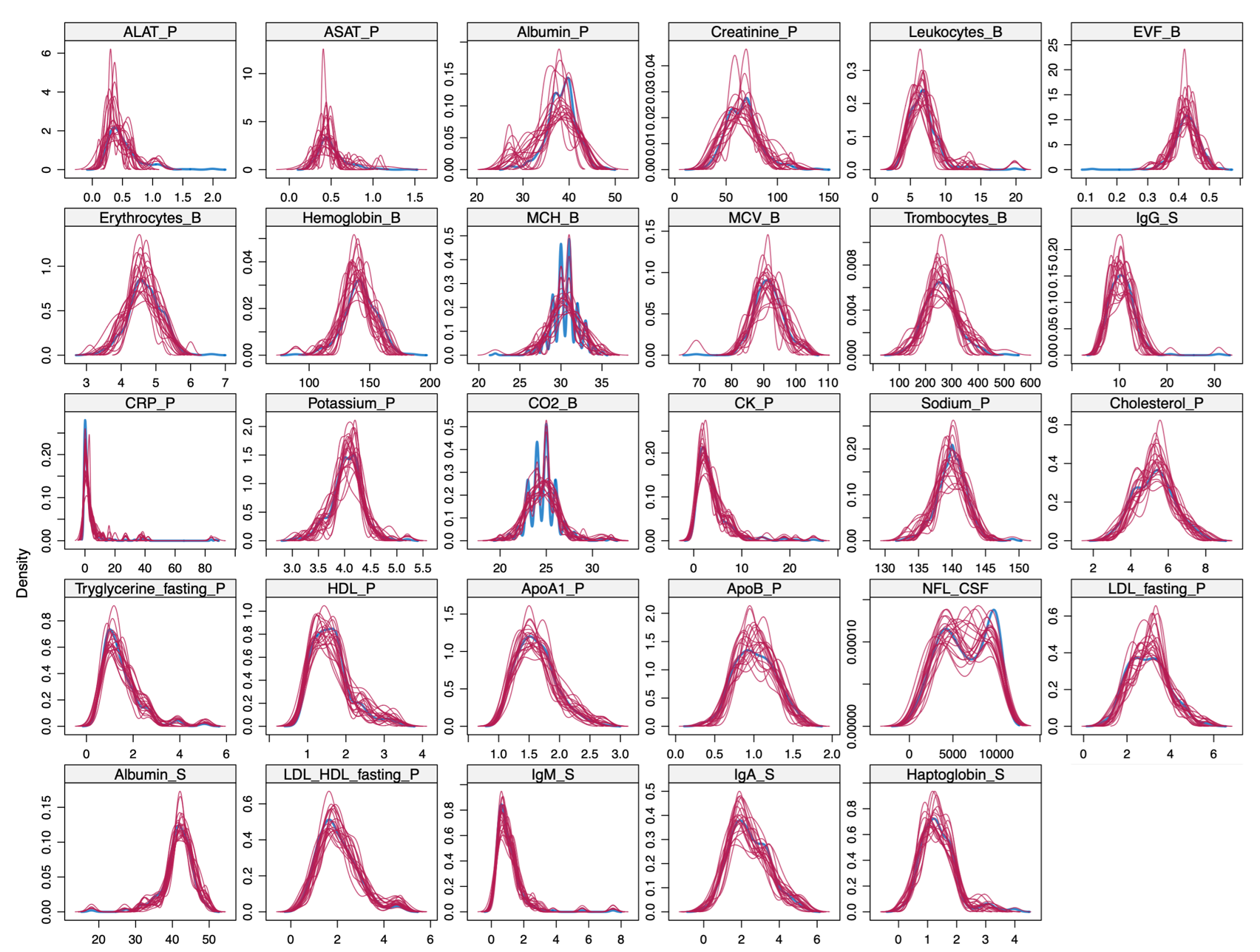


**Supplementary Fig. 1** Density plot of the original (blue curve) and the imputed distributions (red curves) of each biomarker after multiple imputation

**Supplementary Table 1** List of the clinical chemistry biomarkers included in the study, categorized based on their function and the fluid they were derived from

| Category | Biomarkers |
| --- | --- |
| Metabolic | **Plasma**: ALAT, ASAT, Albumin, Creatinine, CRP, Creatinine kinase, Total cholesterol, Triglycerides, HDL, Apolipoprotein A1, Apolipoprotein B, LDL, LDL/HDL ratio  **Serum**: Albumin |
| Hematopoietic | **Whole blood**: Leukocytes, EVF, Erythrocytes, Hemoglobin, MCH, MCV, Thrombocytes |
| Electrolytes | **Whole blood**: CO_2_  **Plasma**: Potassium, Sodium |
| Immunological | **Serum**: IgG, IgM, IgA, Haptoglobin |
| Neurological | **CSF**: NfL |

**Supplementary Table 2** List of all the variables extracted from the Swedish Motor Neuron Disease (MND) Quality Registry

| **Swedish MND register** | **Variables** |
| --- | --- |
| Demographic information | Sex, birth date, death date (where applicable) |
| Clinical information | Diagnosis date, site of symptoms onset, date of symptoms onset, date of initiation of invasive ventilation (where applicable), ALSFRS-R scores, genetic profile, riluzole use, presence of dementia |

**Supplementary Table 3** Associations of biomarkers (per 1-SD increase) with risk of mortality after ALS diagnosis (6 months, 1 year, and 3 years after diagnosis), after multiple imputation, normalization & standardization

| Biomarker | 6 months after diagnosis  (N=270) | | 1 year after diagnosis  (N=270) | | 3 years after diagnosis  (N=270) | |
| --- | --- | --- | --- | --- | --- | --- |
|  | **HR (95% CI)**  **unadjusted^a^** | **HR (95% CI)**  **adjusted^b^** | **HR (95% CI)**  **unadjusted^a^** | **HR (95% CI)**  **adjusted^b^** | **HR (95% CI)**  **unadjusted^a^** | **HR (95% CI)**  **adjusted^b^** |
| Plasma derived |  |  |  |  |  |  |
| ALAT | 0.93 (0.63-1.36) | 1.22 (0.81-1.85) | 1.08 (0.86-1.36) | 1.29 (0.99-1.65) | 0.92 (0.79-1.08) | 1.08 (0.92-1.28) |
| ASAT | 1.14 (0.80-1.61) | 1.31 (0.92-1.88) | 1.08 (0.86-1.36) | 1.12 (0.89-1.42) | 1.04 (0.90-1.21) | 1.07 (0.92-1.23) |
| Albumin | 0.77 (0.53-1.12) | 1.29 (0.86-1.95) | 0.76 (0.60-0.96) | 1.02 (0.79-1.34) | 0.81 (0.70-0.95) | 1.05 (0.88-1.25) |
| C-reactive protein | 0.88 (0.61-1.28) | 0.78 (0.53-1.14) | 1.18 (0.95-1.47) | 1.13 (0.91-1.41) | 1.11 (0.96-1.28) | 1.07 (0.92-1.24) |
| Potassium | 1.03 (0.69-1.54) | 1.11 (0.72-1.70) | 1.07 (0.84-1.36) | 1.08 (0.84-1.39) | 1.01 (0.86-1.19) | 1.00 (0.85-1.19) |
| Sodium | 1.34 (0.77-2.44) | 1.09 (0.61-1.92) | 1.07 (0.73-1.56) | 1.05 (0.73-1.49) | 0.93 (0.72-1.20) | 0.89 (0.70-1.14) |
| Creatinine Kinase | 0.98 (0.66-1.43) | 1.41 (0.88-2.27) | 0.95 (0.74-1.21) | 1.12 (0.86-1.46) | 0.92 (0.79-1.08) | 1.03 (0.87-1.21) |
| Creatinine | 1.19 (0.83-1.69) | 1.23 (0.84-1.81) | 1.05 (0.82-1.33) | 0.98 (0.75-1.28) | 0.98 (0.85-1.15) | 0.93 (0.78-1.11) |
| Total Cholesterol | 0.62 (0.41-0.94) | 0.92 (0.59-1.43) | 0.81 (0.63-1.05) | 1.02 (0.79-1.33) | 0.94 (0.79-1.10) | 1.03 (0.86-1.23) |
| Triglycerides | 0.83 (0.55-1.25) | 0.98 (0.65-1.49) | 0.93 (0.73-1.20) | 1.01 (0.78-1.31) | 0.94 (0.80-1.09) | 0.97 (0.82-1.14) |
| HDL-C | 1.04 (0.71-1.53) | 1.11 (0.69-1.78) | 1.06 (0.83-1.36) | 1.19 (0.89-1.58) | 1.04 (0.89-1.21) | 1.05 (0.88-1.25) |
| LDL-C | 0.57 (0.38-0.87) | 0.86 (0.56-1.32) | 0.76 (0.59-0.99) | 0.95 (0.74-1.22) | 0.93 (0.79-1.10) | 1.03 (0.86-1.22) |
| LDL-C/HDL-C ratio | 0.66 (0.42-1.02) | 0.92 (0.58-1.46) | 0.77 (0.59-1.01) | 0.90 (0.68-1.19) | 0.92 (0.79-1.08) | 1.03 (0.87-1.22) |
| Apolipoprotein A1 | 0.80 (0.51-1.24) | 1.01 (0.61-1.66) | 0.94 (0.72-1.22) | 1.09 (0.80-1.50) | 0.99 (0.85-1.16) | 1.03 (0.86-1.23) |
| Apolipoprotein B | 0.64 (0.43-0.96) | 0.91 (0.62-1.34) | 0.83 (0.65-1.07) | 1.00 (0.79-1.27) | 0.99 (0.84-1.16) | 1.07 (0.91-1.27) |
| Serum derived |  |  |  |  |  |  |
| IgG | 0.76 (0.49-1.17) | 0.90 (0.57-1.42) | 0.85 (0.65-1.11) | 0.93 (0.71-1.21) | 0.93 (0.79-1.09) | 0.93 (0.78-1.10) |
| IgM | 0.94 (0.64-1.40) | 0.99 (0.67-1.46) | 0.86 (0.65-1.13) | 0.91 (0.69-1.20) | 0.92 (0.77-1.10) | 0.95 (0.80-1.13) |
| IgA | 1.09 (0.74-1.61) | 1.24 (0.77-2.01) | 1.14 (0.88-1.47) | 1.17 (0.87-1.59) | 1.08 (0.92-1.27) | 1.08 (0.90-1.29) |
| Haptoglobin | 1.07 (0.74-1.56) | 0.89 (0.61-1.30) | 1.16 (0.92-1.47) | 1.05 (0.82-1.34) | 1.11 (0.94-1.30) | 1.05 (0.89-1.23) |
| Albumin | 0.75 (0.52-1.08) | 1.31 (0.82-2.10) | 0.74 (0.58-0.95) | 1.00 (0.75-1.34) | 0.82 (0.69-0.96) | 0.99 (0.81-1.20) |
| Whole blood |  |  |  |  |  |  |
| Leukocytes | 1.21 (0.88-1.68) | 0.98 (0.70-1.37) | 1.24 (1.02-1.52) | 1.07 (0.87-1.31) | 1.19 (1.02-1.38) | 1.04 (0.90-1.21) |
| EVF | 1.11 (0.76-1.63) | 1.70 (1.09-2.65) | 1.10 (0.86-1.43) | 1.39 (1.03-1.88) | 1.01 (0.85-1.19) | 1.18 (0.97-1.42) |
| Erythrocytes | 0.89 (0.61-1.32) | 1.53 (1.01-2.32) | 0.89 (0.69-1.15) | 1.17 (0.88-1.55) | 0.87 (0.74-1.02) | 1.05 (0.89-1.24) |
| Hemoglobin | 1.09 (0.75-1.59) | 2.07 (1.28-3.37) | 1.08 (0.84-1.39) | 1.45 (1.06-1.97) | 1.00 (0.85-1.17) | 1.20 (0.99-1.46) |
| MCH | 1.24 (0.85-1.80) | 1.03 (0.68-1.56) | 1.35 (1.06-1.71) | 1.18 (0.92-1.52) | 1.26 (1.08-1.46) | 1.12 (0.96-1.31) |
| MCV | 1.53 (1.07-2.19) | 1.16 (0.79-1.70) | 1.50 (1.18-1.89) | 1.30 (1.00-1.69) | 1.33 (1.14-1.56) | 1.12 (0.94-1.33) |
| Thrombocytes | 1.17 (0.82-1.66) | 1.13 (0.76-1.70) | 1.02 (0.81-1.28) | 1.09 (0.86-1.38) | 0.95 (0.82-1.11) | 0.97 (0.83-1.13) |
| CO_2_ | 1.42 (1.08-1.87) | 1.07 (0.79-1.45) | 1.35 (1.11-1.63) | 1.11 (0.91-1.37) | 1.23 (1.05-1.45) | 1.06 (0.90-1.25) |
| CSF derived |  |  |  |  |  |  |
| NFL | 2.61 (1.47-4.65) | 2.19 (1.26-3.80) | 2.27 (1.67-3.10) | 2.00 (1.46-2.73) | 1.57 (1.33-1.85) | 1.48 (1.24-1.75) |

Derived from Cox proportional hazards model
^a^Unadjusted for other covariates
^b^Adjusted for sex, age at diagnosis, onset site, ALSFRS-R score at diagnosis, BMI at diagnosis, and diagnostic delay

**Supplementary Table 4** Associations of EFA-derived factors (per 1-SD increase) with risk of mortality after ALS diagnosis (6 months, 1 year, and 3 years after diagnosis), additionally adjusted for genetic mutation, presence of dementia at the time of diagnosis, and riluzole use at the time of measurement for clinical chemistry markers

| Factor | 6 months after diagnosis^i^  (N=270) | 1 year after diagnosis^i^  (N=270) | 3 years after diagnosis^i^  (N=270) |
| --- | --- | --- | --- |
|  | **HR (95% CI)**  **Adjusted^a^** | **HR (95% CI)**  **Adjusted^a^** | **HR (95% CI)**  **Adjusted^a^** |
| LDL Profile | 0.80 (0.50-1.30) | 0.92 (0.70-1.20) | 1.01 (0.84-1.21) |
| Red Blood Cell Profile | 1.70 (1.05-2.77) | 1.42 (1.05-1.93) | 1.23 (1.00-1.52) |
| HDL Profile | 1.04 (0.60-1.80) | 1.17 (0.86-1.60) | 1.05 (0.87-1.28) |
| Liver enzymes & CK | 1.47 (0.95-2.28) | 1.25 (0.97-1.61) | 1.14 (0.98-1.33) |
| Albumin & CRP | 1.44 (0.88-2.35) | 0.98 (0.74-1.28) | 0.98 (0.82-1.17) |
| Hematological Profile | 0.99 (0.66-1.48) | 1.09 (0.89-1.34) | 1.03 (0.89-1.19) |

^i^Derived from multivariable Cox proportional hazards model
^a^Adjusted for sex, age at diagnosis, onset site, ALSFRS-R score at diagnosis, BMI at diagnosis, diagnostic delay, genetic mutation, presence of dementia at the time of diagnosis, and riluzole use at the time of measurement for clinical chemistry markers.

**Supplementary Table 5** Associations of the six factors with the probability of belonging to different ALSFRS-R trajectories after excluding patients with a probability of belonging to a class as ≤ 0.7 (N = 222)

| Factor | OR (95% CI)  Unadjusted | OR (95% CI)  Adjusted^i^ | Factor | OR (95% CI)  Unadjusted | OR (95% CI)  Adjusted^i^ |
| --- | --- | --- | --- | --- | --- |
| LDL Profile |  |  | **Liver enzymes & CK** |  |  |
| Slow | 0.95 (0.62-1.46) | 0.85 (0.52-1.42) | Slow | 1.02 (0.70-1.50) | 1.11 (0.71-1.74) |
| Intermediate | 1.22 (0.87-1.70) | 1.12 (0.78-1.61) | Intermediate | 0.94 (0.68-1.29) | 1.02 (0.73-1.43) |
| Fast (Ref.) | - | - | Fast (Ref.) | - | - |
| Red Blood Cell Profile |  |  | **Albumin & CRP** |  |  |
| Slow | 0.81 (0.54-1.22) | 0.75 (0.44-1.28) | Slow | 1.09 (0.73-1.64) | 0.95 (0.55-1.65) |
| Intermediate | 0.83 (0.60-1.15) | 0.85 (0.58-1.26) | Intermediate | 1.12 (0.81-1.55) | 1.00 (0.69-1.46) |
| Fast (Ref.) | - | - | Fast (Ref.) | - | - |
| HDL Profile |  |  | **Hematological Profile** |  |  |
| Slow | 0.87 (0.58-1.30) | 0.78 (0.47-1.32) | Slow | 0.77 (0.49-1.21) | 0.85 (0.50-1.43) |
| Intermediate | 0.94 (0.68-1.30) | 0.83 (0.57-1.20) | Intermediate | 1.12 (0.82-1.54) | 1.12 (0.80-1.55) |
| Fast (Ref.) | - | - | Fast (Ref.) | - | - |

Derived from multinomial logistic regression. All factors were included in the same model

^i^Adjusted for sex, age at diagnosis, onset site, ALSFRS-R score at diagnosis, BMI at diagnosis, and diagnostic delay

**Supplementary Table 6.** Associations of baseline neurofilament light chain (NfL) in cerebrospinal fluid (CSF) with the probability of belonging to different ALSFRS-R trajectories after excluding patients with a probability of belonging to a class as ≤ 0.7 (N = 222)

| NfL in CSF | OR (95% CI)  Unadjusted | OR (95% CI)  Adjusted^i^ |
| --- | --- | --- |
| Slow | 0.26 (0.16-0.44) | 0.29 (0.16-0.52) |
| Intermediate | 0.39 (0.26-0.59) | 0.42 (0.27-0.65) |
| Fast (Ref.) | - | - |

Derived from multinomial logistic regression.

^i^Adjusted for sex, age at diagnosis, onset site, ALSFRS-R score at diagnosis, BMI at diagnosis, and diagnostic delay
